# Maximizing efficiency of genomic selection in CIMMYT’s tropical maize breeding program

**DOI:** 10.1007/s00122-020-03696-9

**Published:** 2020-10-10

**Authors:** Sikiru Adeniyi Atanda, Michael Olsen, Juan Burgueño, Jose Crossa, Daniel Dzidzienyo, Yoseph Beyene, Manje Gowda, Kate Dreher, Xuecai Zhang, Boddupalli M. Prasanna, Pangirayi Tongoona, Eric Yirenkyi Danquah, Gbadebo Olaoye, Kelly R. Robbins

**Affiliations:** 1 West Africa Center for Crop Improvement (WACCI), University of Ghana, Accra, Ghana; 2 International Maize and Wheat Improvement Center (CIMMYT), Texcoco, Mexico; 3 Section of Plant Breeding and Genetics, School of Integrative Plant Sciences, Cornell University, Ithaca, NY USA; 4 International Maize and Wheat Improvement Center (CIMMYT), Nairobi, Kenya; 5 Agronomy Department, University of Ilorin, Ilorin, Nigeria

## Abstract

**Key message:**

Historical data from breeding programs can be efficiently used to improve genomic selection accuracy, especially when the training set is optimized to subset individuals most informative of the target testing set.

**Abstract:**

The current strategy for large-scale implementation of genomic selection (GS) at the International Maize and Wheat Improvement Center (CIMMYT) global maize breeding program has been to train models using information from full-sibs in a “test-half-predict-half approach.” Although effective, this approach has limitations, as it requires large full-sib populations and limits the ability to shorten variety testing and breeding cycle times. The primary objective of this study was to identify optimal experimental and training set designs to maximize prediction accuracy of GS in CIMMYT’s maize breeding programs. Training set (TS) design strategies were evaluated to determine the most efficient use of phenotypic data collected on relatives for genomic prediction (GP) using datasets containing 849 (DS1) and 1389 (DS2) DH-lines evaluated as testcrosses in 2017 and 2018, respectively. Our results show there is merit in the use of multiple bi-parental populations as TS when selected using algorithms to maximize relatedness between the training and prediction sets. In a breeding program where relevant past breeding information is not readily available, the phenotyping expenditure can be spread across connected bi-parental populations by phenotyping only a small number of lines from each population. This significantly improves prediction accuracy compared to within-population prediction, especially when the TS for within full-sib prediction is small. Finally, we demonstrate that prediction accuracy in either sparse testing or “test-half-predict-half” can further be improved by optimizing which lines are planted for phenotyping and which lines are to be only genotyped for advancement based on GP.

**Electronic supplementary material:**

The online version of this article (10.1007/s00122-020-03696-9) contains supplementary material, which is available to authorized users.

## Introduction

The Food and Agriculture Organization (FAO) estimates that by 2050 the world’s population will surpass 9 billion people (Nations and United Nations 2019). Much of this population growth will occur in regions of the world where food insecurity is prevalent, with large increases in food demand projected in Sub-Saharan Africa (SSA) and South Asia (SA). While improving food security in SSA and SA requires a multi-faceted approach, accelerating genetic gains and enhancing performance of crop varieties in smallholder farmers’ fields is vital. National and international research organizations continue to play an important role in crop improvement in SSA and SA, although investment in the public-sector breeding programs is significantly lower relative to countries in other regions of the world (Kremer and Zwane 2005; Langyintuo et al. 2010; Kremer and Zwane 2005). Since scaling up the size of the public-sector breeding programs is not feasible, new approaches are required to significantly increase the rate of genetic gain.

A core goal of CIMMYT’s maize and wheat breeding programs is to respond to the present and emerging challenges by developing and deploying improved varieties particularly for the benefit of resource-constrained smallholder farmers who operate in challenging environments. Advances in the use of genomic information in crop breeding programs have the potential to significantly increase genetic gains. Genomic selection (GS) is a breeding method where the performance of new breeding materials is predicted based on genomic information (Meuwissen et al. 2001). Multiple studies have shown the potential of this methodology to increase the rates of genetic gain in breeding programs by reducing the cost and time associated with extensive phenotyping of new offspring to identify the best performers for use as parents in the next generation (de los Campos et al. 2010; Crossa et al. 2010, 2011, 2017; Lin et al. 2014; Hickey et al. 2017; Beyene et al. 2019). GS can improve breeding program efficiency if properly designed and implemented to fully harness and maximize its advantages.

As genotyping costs have significantly declined relative to phenotyping costs in recent time, GS has become an attractive option as a selection decision tool in breeding programs. The CIMMYT Global Maize Program has been evaluating various strategies to implement GS in the first year testcross trial stage with an objective of selecting promising lines for advancement into more resource intensive multi-tester, multi-location yield trials (Beyene et al. 2015, 2019; Vivek et al. 2017). Efficient identification of superior lines at this early stage also enables recycling of lines back as breeding parents, thereby decreasing the breeding cycle time.

Phenotyping is required to calibrate models for predicting genomic estimated breeding values (GEBV) of untested genotypes, meaning that in a breeding program utilizing GS, data collected from field testing are no longer used solely for the purpose of informing immediate selection decisions. To achieve adequate genomic prediction accuracy, the population of individuals phenotyped must be related to the breeding population and the testing environments must be correlated with the target population of environments (Burgueño et al. 2012; Jarquín et al. 2014; Santantonio et al. 2020).

**Fig. 1:**
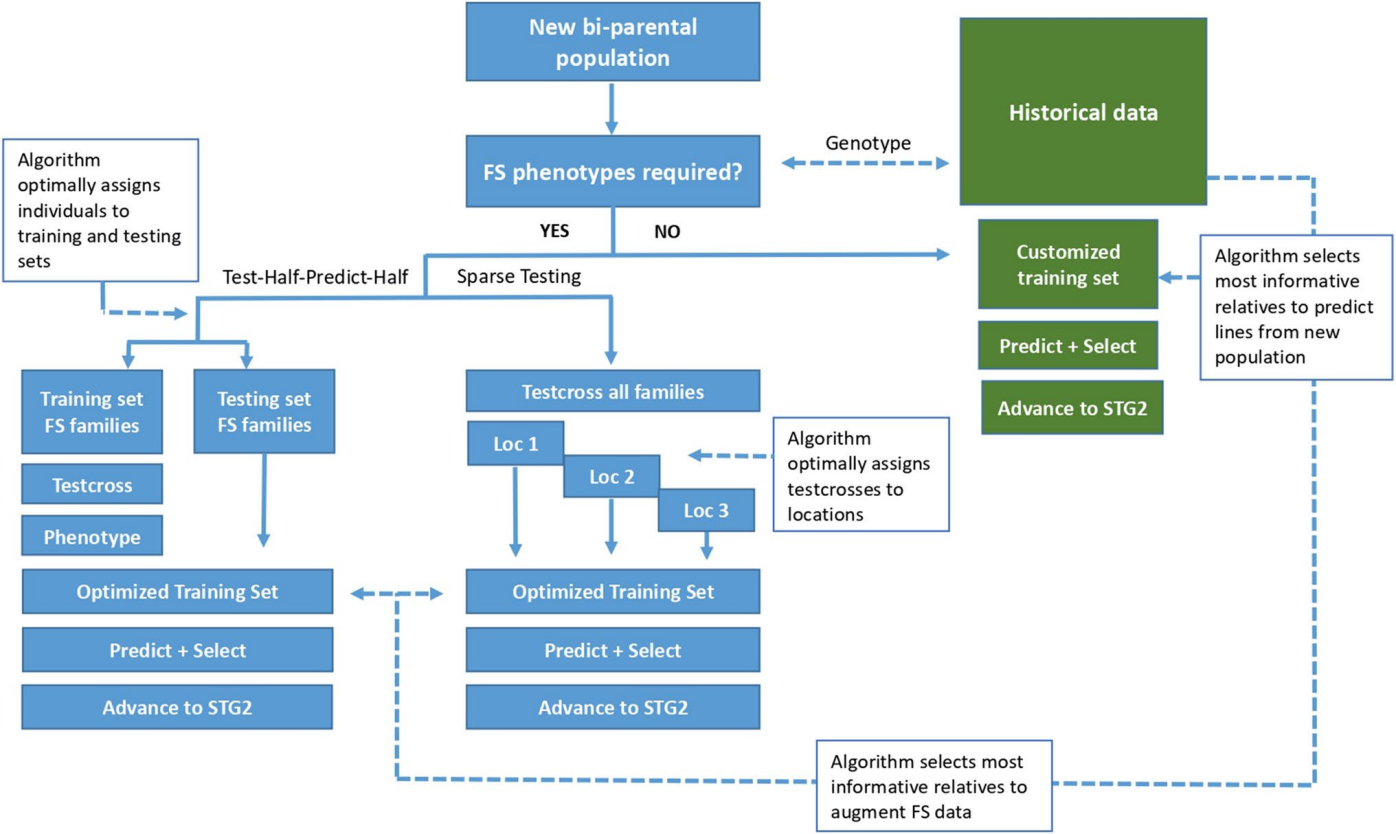
Schematic illustration of the stepwise approach for improving prediction accuracy and maximizing advantages of genomic selection in CIMMYT’s tropical maize breeding program. The strategy proposes the use of algorithms to select individuals from historical data to achieve high predictive performance for new populations and enable advancement into stage 2 yield trials. When historical data are not sufficient to predict the performance of new lines, test-half-predict-half and sparse testing approaches are used

The CIMMYT maize breeding strategy is evolving from a phenotype only-based system to a GS-based system in a phased manner (Santantonio et al. 2020). In the current phase, a test-half-predict-half (THPH) strategy is being implemented which involves testing half of the full-sib lines within each bi-parental population and including data from the phenotyped full-sib testcrosses together with other testcross information from within the same heterotic group. The approach has been adopted for each specific product profile-based tropical breeding program to form training sets to predict the testcross performance of untested lines (Beyene et al. 2015, 2019; Zhang et al. 2015). Although this strategy has proven effective in improving the cost efficiency of the breeding programs, the goal is to adapt approaches for the use of across-year and across-breeding programs data to further improve the selection accuracy of GS, to optimize resource use efficiency within the tropical breeding programs of CIMMYT, and to eventually reduce the length of variety testing and breeding cycle by eliminating the need for the first year yield testing (Fig. 1).

As part of the phased GS implementation strategy, the THPH approach has been implemented across all tropical and Latin America breeding programs for the last two to three years while a robust historical training dataset is developed for each specific breeding program to enable prediction directly into stage 2 yield trials. To more effectively implement the longer term strategy, methods are being investigated to inform model training decisions on a population by population basis using accumulated historical data across multiple tropical programs of CIMMYT. To efficiently implement this approach, algorithms are being evaluated to optimally extract the most informative training set for predicting a given population (Fig. 1).

Ideally, the algorithms used would first inform decisions on whether a new untested population has sufficient close relatives in the historical data set or if it would require some full-sib testcross phenotype data from a stage 1 trial to provide adequate confidence to advance lines from the population to stage 2. Following decisions on whether to predict and select directly into stage 2 or to employ some form of GS assisted stage 1 testing (either THPH, sparse testing (ST), or a combination of the two). Algorithms to extract the most informative training set on a population by population basis would enable maximization of prediction accuracy across a diverse set of breeding programs that share some common founder lines. To enable the next phase of GS implementation in CIMMYT’s tropical maize breeding programs, several methods to optimally extract customized training sets (CTS) on a population by population basis have been evaluated and compared using cross-validation conducted using data collected in 2017 and 2018.

Two optimization criteria: (a) generalized coefficient of determination (CD) as proposed by Laloë (1993) and Rincent et al. (2012), and (b) average genomic relationship (Avg_GRM) between each individual of training population (TP) and target breeding population (BP) were evaluated for selecting subsets of individuals with good predictive ability for each target population. The objectives of the study were to (1) determine the best method for selecting customized training sets; (2) ascertain if the customized training sets improved the prediction accuracy when compared to using all of the diverse populations for model calibration; (3) recommend optimal resource allocation where phenotyping of full-sib families with half-sib pedigree relationship is required; and (iv) determine the impact of using algorithms such as CDmean to determine which lines to phenotype in which environments in both ST and THPH genomic selection strategies without increasing breeding expenditure. Overall, this study will further contribute to the existing body of literature on the importance of training population design and genomic relationship between TP and BP on prediction accuracy.

## Materials and methods

### Phenotypic data

The phenotypic data consist of doubled haploid (DH) maize lines from CIMMYT’s eastern Africa maize breeding program at preliminary/stage 1 yield trials evaluated in Kiboko (Lat. 2° 15′ S, Long. 37° 75′ E, approximately 975 above sea level) and Kakamega (Lat. 0° 16′ N, Long. 34° 49′ E, approximately 1585 above sea level) in Kenya during the 2017 and 2018 growing seasons, respectively. Herein, we refer to these 2017 and 2018 datasets as DS1 and DS2, respectively. The DH lines, which do not overlap across years, were testcrossed to three single cross testers in DS1 and two single cross testers in DS2, respectively. Information regarding the 849 DH lines and 1389 DH lines is provided (Online Resources 1 and 2).

The testcrosses were evaluated in 13 trials in DS1 and 34 trials in DS2. The trials in each dataset were connected by common checks. The checks and the testcrosses in each trial were planted in an alpha-lattice incomplete block design with two replications in each location. The entries were planted two-rows per plot, each row was 5 m long, with spacing of 0.75 m between rows and 0.25 m between hills. At planting, two seeds per hill were planted and thinned to one plant per hill three weeks after emergence to obtain a final plant population density of 53,333 plants per hectare. Fertilizers were applied at the rate of 60 kg N and 60 kg P_2_O_5_ per ha, as recommended for the area. Nitrogen was applied in a split dose at planting and 6 weeks after emergence.

Grain yield and other agronomic traits were recorded per trial at each location but only grain yield (GY) was reported in this study. To mimic historical data, DS1 and DS2 were combined and optimization algorithms (CDmean and Avg_GRM) were used for the selection of individuals in the combined data that were most informative of a given BP. The prediction accuracy of customized training sets was compared with the prediction accuracy obtained using the entire data set. The genetic optimization criteria are discussed further in “Optimization criteria” section.

### Genotypic data

All DH lines were genotyped using repeat Amplification Sequencing (rAmpSeq) at Cornell Life Science Core Laboratory Center, Ithaca, NY, USA. The rAmpSeq genotyping platform makes use of knowledge of whole-genome sequences and repetitive sequences to identify DNA sequence polymorphisms using novel bioinformatics tools (Buckler et al. 2016). The rAmpSeq platform provides dominant markers, with the 9155 sequence tags coded as 0 and 2 based on presence or absence of the marker, respectively. The 6785 markers with minor allele frequency greater than 0.05 were used for analysis.

### Statistical analysis

#### Phenotypic analysis

A linear mixed model was fit using ASREML-R (Butler et al. 2017; R Core Team 2019) for combined trial analysis and across locations in DS1 and analysis of the combined DS1 and DS2 to estimate best linear unbiased estimate (BLUE) for each line:

1 $${\mathbf{y}} = 1_{n} \mu + {\mathbf{X}}_{1} {\mathbf{b}}_{1} + {\mathbf{X}}_{2} {\mathbf{b}}_{2} + {\mathbf{Z}}_{1} {\mathbf{u}}_{1} + {\mathbf{Z}}_{2} {\mathbf{u}}_{2} + {\mathbf{Z}}_{3} {\mathbf{u}}_{3} + {\mathbf{Z}}_{4} {\mathbf{u}}_{4} + {\varvec{\upvarepsilon}}$$

and

2 $${\mathbf{y}} = 1_{n} \mu + {\mathbf{X}}_{1} {\mathbf{b}}_{1} + {\mathbf{X}}_{2} {\mathbf{b}}_{2} + {\mathbf{Z}}_{1} {\mathbf{u}}_{1} + {\mathbf{Z}}_{2} {\mathbf{u}}_{2} + {\mathbf{Z}}_{3} {\mathbf{u}}_{3} + {\mathbf{Z}}_{4.1} {\mathbf{u}}_{4.1} + {\varvec{\upvarepsilon}}$$

While the BLUE of the lines in each location was calculated using the model below:

3 $${\mathbf{y}} = 1_{n} \mu + {\mathbf{X}}_{1} {\mathbf{b}}_{1} + {\mathbf{X}}_{2.1} {\mathbf{b}}_{2.1} + {\mathbf{Z}}_{1} {\mathbf{u}}_{1} + {\mathbf{Z}}_{2} {\mathbf{u}}_{2} + {\mathbf{Z}}_{3.1} {\mathbf{u}}_{3.1} + {\varvec{\upvarepsilon}}$$

where **y** (*n *× 1) is the vector of phenotypes for each DH lines, *μ* is the overall mean, $1_{n}$(*n *× 1) is a of vector ones, ${\mathbf{b}}_{1}$ is the fixed effect of the line, ${\mathbf{b}}_{2}$ is the fixed effect of the location, ${\mathbf{b}}_{2.1}$ is the fixed effect of replication, ${\mathbf{u}}_{1}$ is the random effect of the trial, ${\mathbf{u}}_{2}$ is the random effect of the tester, ${\mathbf{u}}_{3}$ is the random effect of genotype by location interaction, ${\mathbf{u}}_{3.1}$ is the random effect of block within replication, ${\mathbf{u}}_{4}$ is the random effect of incomplete block nested within replication, trial and location, ${\mathbf{u}}_{4.1}$ is the random effect of incomplete block by replication by trial by location by year. ${\mathbf{X}}_{n}$ and ${\mathbf{Z}}_{m}$ are the incidence matrices for fixed and random effects, respectively. The *n* and *m* represent the number of fixed and random effects.

The variance of the random effects ($u_{1}$, $u_{2}$, $u_{3}$, $u_{3.1}$, $u_{4}$, $u_{4.1}$) were assumed to be distributed as:

$$u_{m} \sim N\left( {0, {\mathbf{I}}\sigma_{{u_{m} }}^{2} } \right)$$

where **I** is the identity matrix.

The adjusted mean for each DH line was estimated as the sum of the population mean with the fixed genetic effect of the *i*-th line that is $\hat{y} = \hat{\mu } + \hat{b}_{i}$. Broad sense heritability was calculated from the variance components obtained by refitting the models with all terms as random effects using restricted maximum likelihood (REML) as:

$$\frac{{\sigma_{g}^{2} }}{{\sigma_{g}^{2} + \frac{{\sigma_{gl}^{2} }}{{n_{l} }} + \frac{{\sigma_{\varepsilon }^{2} }}{{n_{l} n_{r} }}}}\;{\text{and}}\;\frac{{\sigma_{g}^{2} }}{{\sigma_{g}^{2} + \frac{{\sigma_{gl}^{2} }}{{n_{l} }} + \frac{{\sigma_{\varepsilon }^{2} }}{{n_{l} n_{k} n_{r} }}}}$$

where $\sigma_{g}^{2}$ is the genetic variance, $\sigma_{gl}^{2 }$ is the genotype by location interaction variance and $\sigma_{\varepsilon }^{2}$ is the residual variance, *n*_l_, *n*_*r*_ and *n*_*k*_ are the number of locations, replicates and years.

#### Estimation of genomic relationship

Using the (VanRaden 2008) equation, the genomic relationship on DH lines was estimated as below:

4 $${\mathbf{G}}_{{{\mathbf{DH}}}} = \frac{{{\mathbf{WW^{\prime}}}}}{{2\sum p_{j} \left( {1 - p_{j} } \right)}}$$

Elements of matrix **W** are $w_{ij}$ where $w_{ij}$ is the genotype represented as the number of copies of the dominant allele of DH line *i* at marker *j,* denoted as 0 or 2 for the recessive and dominant homozygous, respectively, and $p_{j}$ is the allele frequency at marker *j*. Given the lines were DH, it was assumed all lines with the carrying the dominant marker were homozygous dominant. The denominator is calculated such that the expectation of the genomic relationship matrix is the numerator relationship matrix (VanRaden 2008). Given the inbreds were tested as parents of hybrids, the genomic relationship used to calculated GCAs was as follows:

$${\mathbf{G}} = .5*{\mathbf{G}}_{{{\mathbf{DH}}}}$$

#### Genomic selection model

The ST genomic selection strategy is an unbalanced design in which half of the population was planted in one location and the other half in another location (Burgueño et al. 2012; Jarquín et al. 2014; Santantonio et al. 2020). This takes advantage of allele replication across the locations even though the lines were not replicated across locations. The GEBV of the testing set in both ST and THPH genomic selection strategies was estimated using an unstructured covariance model which is an extension of the genomic best linear unbiased prediction (GBLUP) mixed model that allows borrowing of information from genetically correlated locations. This model was fit using the average information algorithm of ASReml (Gilmour et al. 1995). Generally, the GBLUP model can be expressed as:

5 $${\mathbf{y}} = {\mathbf{X\beta }} + {\mathbf{Zu}} + {\varvec{\upvarepsilon}}$$

where **y** is a vector of plot observation in each location and for other cross-validation schemes it is the BLUE of each DH lines, **β** is a vector of fixed effect, **u** is a vector of genomic breeding values (GEBV), **X** and **Z** are design matrices, and **ε** is the vector of residuals. The variance of **u** is assumed to be distributed as

$${\mathbf{u}} \sim N(0, {\mathbf{G}} \otimes {\text{cov(}}{\mathbf{E}}_{ij} ) )$$

where ${\mathbf{G}} \otimes {\text{cov}}\left( {{\mathbf{E}}_{ij} } \right)$ is the kronecker product between the genomic relationship of the lines (**G)** and the genetic (co)variance of GEBVs within and across locations (${\text{cov}}\left( {{\mathbf{E}}_{ij} } \right)$). The variance of the residuals were assumed to be

$${\varvec{\upvarepsilon}} \sim N(0,{\text{diag(}}\sigma_{\varepsilon \left( i \right)}^{2} ))$$

where ${\text{diag}}\left( {\sigma_{\varepsilon \left( i \right)}^{2} } \right)$ is a diagonal matrix with different residual variance for each location. Prediction accuracy of unobserved genotypes was determined as the Pearson correlation of the observed BLUEs to the predicted GEBV.

The predicted GEBV (**u**) of untested genotypes within location was correlated with the estimated BLUE for each location (Eq. 3) and the mean was calculated as prediction accuracy across locations.

#### Optimization criteria

The most informative TP for model calibration to predict the genetic merit of BP are the individuals that are closely related to BP. When pairs of individuals are closely related they tend to inherit quantitative trait loci (QTLs) blocks in the same linkage phase (Habier et al. 2010). This is particularly important when the density of markers being used for prediction is low and linkage disequilibrium between markers decays rapidly in less related individuals. Andreescu et al. (2007) reported that LD consistency between SNPs and QTLs across populations is related to the degree of relationship between populations and marker density. Multiple criteria such as effective chromosome segments (*M*_*e*_), deterministic prediction accuracy (DPA), CDmean and Avg_GRM were used to select subsets of TP that are most informative for a given BP. Initial analyses, reported in (Online Resources 3A and B), showed that CDmean and Avg_GRM consistently yielded the best results and thus were selected for further investigation.

#### Coefficient of determination

The coefficient of determination (CD) is the expected reliability of the predicted genetic values of the BP (Laloë 1993; Rincent et al. 2012). The expected reliability of the prediction of the different contrasts was expressed as:

6 $${\text{CDmean}} = {\text{diag}}\left[ {\frac{{{\mathbf{K^{\prime}}}({\mathbf{G}} - \lambda \left( {{\mathbf{Z^{\prime}DZ}} + {\mathbf{\lambda G}}^{ - 1} } \right)^{ - 1} ){\mathbf{K}}}}{{{\mathbf{K^{\prime}GK}}}}} \right]$$

where **D** = **I** − **X(X′X)**^**−1**^ **X′**,**(X′X)**^**−1**^ is the generalized inverse of **X′X**, *λ* = *σ*_ε_^2^/*σ*_g_^2^, *σ*_ε_^2^ is the residual error and *σ*_*g*_^2^ is the genetic variance. The variance component estimates were obtained from either Eq. 1 where the goal was to select CTS within DS2 or Eq. 2 when the goal was to select CTS from harmonized DS1 and DS2. In scenarios investigating the optimization of ST and THPH designs (i.e., partitioning of lines across locations and training/test sets) *λ* was set to 0.5. Our initial analysis (results not shown) showed that the efficiency of CDmean is not highly dependent on trait heritability but rather on genomic relationship. When an intermediate value was chosen for *λ* the prediction accuracy was close to the actual *λ*. This was in agreement with Rincent et al. (2012). While results in this study were robust to values of *λ*, strategies for optimizing *λ* should be further explored. **G**, **X** and **Z** is the same as defined above and **1** **K′** is a contrast vector and the sum of its elements equal to zero. The dominators ${\mathbf{K^{\prime}GK}}$ prevent the selection of closely related individuals in the potential CTS thus selecting individuals that spread through the genetic space of the BP.

In this study, CDmean was used for two optimization objectives: (1) to quantify connectedness and select individuals from multiple bi-parental populations that are closely related to a given BP, and (2) to optimize experimental designs for ST and THPH. Objective 1 was achieved by maximizing the mean of the CD of the contrast between the genetic values of individuals in the BP and the mean of the population (BP + different subset of TP). This is calculated as CDmean = mean [diag(CD(**K**))], where **K** is a matrix of contrasts with dimension (*n*) number of individuals in the population. The CDmean decreases as the relationship between BP and the subset of TP declines and when the relationship of individuals in the subset of TP is larger than their relationship to a target BP. Thus, the greater the mean of the contrast, the more closely related the subset of TP to the BP and the less related are the individuals within the optimized TP. To achieve this the hill-climbing exchange algorithm proposed by (Rincent et al. 2012) was implemented and the number of exchange moves was set to 8000. The algorithm starts with a random size (*n*) of potential CTS which is used to calculate the initial CDmean. At each move, one random individual was removed from the initial set, replaced with another random individual from the remaining TP, the move is accepted if the CDmean increases and rejected if otherwise. For objective 2, groups of DH lines were selected to maximally represent the genetic space of each bi-parental family. Following Santantonio et al. (2020), CDmean was used for training set design in both THPH and STGS strategies by optimizing selection of individuals phenotyped (THPH) and planted in each environment (ST).

#### Average genomic relationship

Average genomic relationship (Avg_GRM) between a specific line in the TP and all lines in the target BP was calculated for all individuals in the TP. We assumed that Avg_GRM is a raw estimate of the proportion of the genome shared between potential CTS and all individuals in the BP. Thus, the probability of selecting an individual as part of the CTS depends on its high Avg_GRM value with the BP.

7 $${\text{Avg}}\_{\text{GRM}}_{j} = \frac{1}{N}\mathop \sum \limits_{i}^{N} {\mathbf{G}}_{ij}$$

where ${\mathbf{G}}_{ij}$ is the realized kinship coefficient between the *i*th individual in a target BP and the *j*th line in the TP and *N* is the size of target BP.

#### Cross-validation scheme

The prediction accuracy of a target population obtained from models calibrated with different (*n*) subsets of individuals selected from DS2 using CDmean and Avg_GRM was compared to prediction accuracy from an average of 50 random selections of different subset sizes as above, using half-sib related populations and all the DS2 as TP. Prediction accuracies were calculated as the Pearson correlation of the predicted GEBV and the BLUE estimates of DH lines in DS2 obtained from Eq. 1. Six families with population size ≥ 49 were exclusively considered for use as target population (Online Resources 2). DS1 and DS2 were combined to further evaluate the efficiency of the algorithms and the impact on prediction accuracy when the training population has individuals with varying degree of pedigree relationship. The prediction accuracies obtained using optimized TP were calculated as the Pearson correlation of the predicted GEBV and BLUE estimates of DH lines obtained from the combined analysis (Eq. 2). Finally, the prediction accuracy of TP design in ST and THPH using CDmean was compared to the average accuracy from 50 random splits using DS1. The predicted GEBV of untested DH lines within location was correlated with the estimated BLUE for each location (Eq. 3) and averaged across locations.

In addition, we explored the most efficient strategy for the use of half-sib related populations for training set development in GS using DS1. For this, only 8 families with population size ≥ 63 were considered (Online Resources 1). In the first scenario, each population was randomly partitioned into 50, 70, 80, or 90% of the testing set and the remaining 50, 30, 20, or 10% from all the populations, excluding the testing population, was collectively used as a training set so that no individuals from the target BP were included in the training set. For the second scenario, populations were randomly split as above but the training set included part of the target testing population. As a baseline for comparison, within full-sib (WFS) prediction was conducted for each population by randomly splitting the populations in the opposite direction of the first two strategies that is 50, 30, 20, or 10% as testing set and 50,70, 80, or 90% as training set. A total of 50 random splits were used to estimate an average cross-validation accuracy. Here, the prediction accuracy was calculated as the Pearson correlation of the estimated GEBV from the model calibrated using the different training set designs and the BLUE estimates from analysis of DS1 using Eq. 3.

## Results

### Population structure

**Fig. 2:**
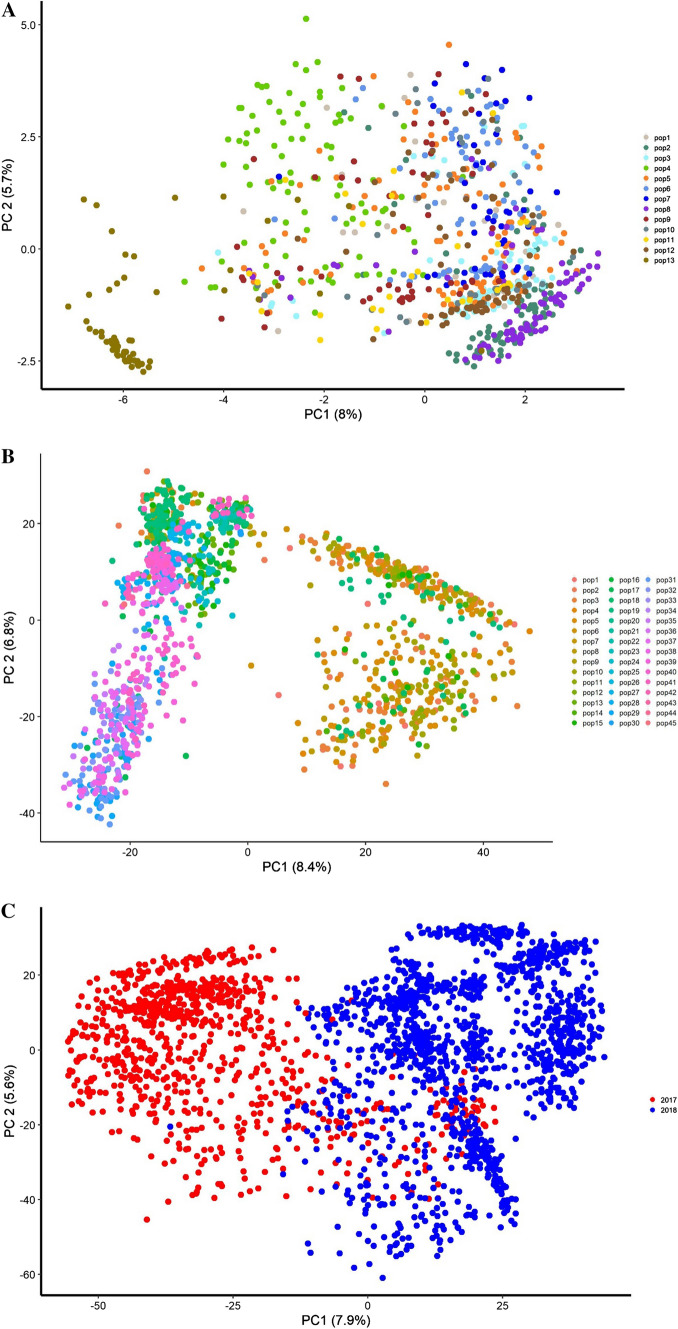
**a** Spectral decomposition of the genomic relationship matrix of DS1 (849 DH lines). The plot of the first two principal components shows the population structure in DS1, each dot represents a DH line and the colors are each bi-parental population. **b** Spectral decomposition of the genomic relationship matrix of DS2 (1389 DH lines). The plot of the first two principal components shows the population structure in DS2, each dot represents a DH line and the colors are each bi-parental population. **c** Spectral decomposition of the genomic relationship matrix of the combined D1 and DS2 (2238 DH lines). The plot of the first two principal components shows the interconnectedness across the datasets. Each dot represents a DH line, and the blue and red colors represent DH lines in DS1 and DS2, respectively

DS1 comprises 13 full-sib families (FSF). Populations 1–12 have one parent in common (La Posta Seq C7-F64-2-6-2-2-B–B) and populations 4 and 13 share CML312 as a common parent (Online Resources 1). The population structure was assessed using principle components calculated from the spectral decomposition of the genomic relationship matrix. The pedigree classification of the dataset was corroborated by the plot of the first two principal components (PCs) with significant overlaps between populations, with the exception of population 13 (Fig. 2a). The 1389 DH lines in DS2 were classified into 45 FSF based on pedigree information (Online Resources 2) and the diversity within the dataset is illustrated by the plot of the PCs (Fig. 2b). The pedigree relationship of some populations across datasets (2017 and 2018) was also explained by the overlap between the two datasets as shown by the plot of the first two principal components (Fig. 2c) of the realized genomic relationship matrix of the 2238 DH lines.

### Efficiency of optimization criteria and predictive ability of multiple Bi-parental populations

**Fig. 3:**
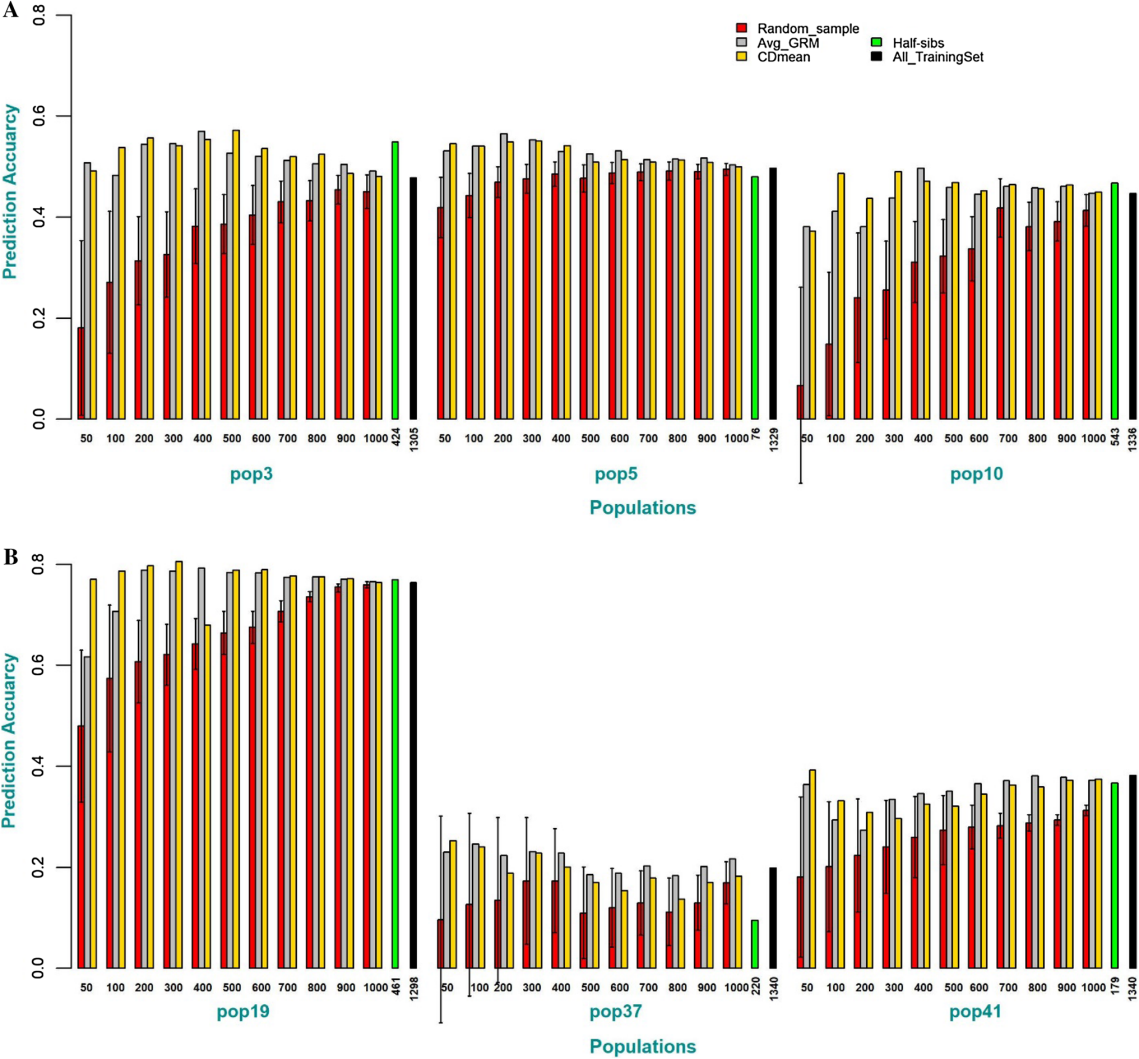
**a** Prediction accuracy of different training set sizes optimized using Avg_GRM and CDmean compared to the random sampling of the training set or the use of all populations (DS2) as a training set for populations 3, 5 and 10. For the random sampling (red color), the error bar represents the degree of variation in prediction accuracy of the 50 repetitions of random selection of individuals as the training set. **b** Prediction accuracy of different training set sizes optimized using Avg_GRM and CDmean compared to the random sampling of the training set or the use of all populations (DS2) as a training set for populations 19, 37 and 41. For the random sampling (red color), the error bar represents the degree of variation in prediction accuracy of the 50 repetitions of random selection of individuals as the training set

Estimates of broad-sense heritability for grain yield in 2017, 2018, and combined 2017 and 2018 datasets were 0.65, 0.69, and 0.60, respectively. The prediction accuracy for training sets optimized using *M*_e_, DAPH and DPAR were better than the mean prediction accuracy of randomly selected training sets, except for populations 5 and 19. However, unlike CDmean and Avg_GRM, when training set sizes were less than 600, the prediction accuracy for training sets optimized using M_e_, DAPH and DPAR did not improve beyond prediction accuracy obtained using all the populations as a training set (Online Resources 3A and 3). Figure 3 illustrates the consistent high prediction accuracy of the predicted populations using CDmean and Avg_GRM to select subset sizes of individuals from the DS2 that are most informative of a target BP for model calibration compared to the random selection of the CTS. The percentage of individuals that have half-sib relationships with target BP selected by the two selection criteria in the different subset sizes (50 to 1000) range from 0.92 to 0.13 depending on the size of the subset and the sum of individuals with half-sib relationship to a target BP present in the DS2 dataset. The number of individuals that have half-sib relationships with each of the target BP ranged from 78 to 504 individuals (Online Resource 1). Thus, for subset sizes of 50 to 500, the percentage of populations with half-sib pedigree relationship in the subsets was ≥ 50 percent, illustrating the efficiency of the selection criteria in selecting individuals related to the BP from the DS2. Selecting individuals from the multiple bi-parental populations that are closely related to a target BP for model training resulted in higher prediction accuracy compared to using all the DS2 as a training set (Table 1).

**Table 1:** The percentage of individuals with half-sib pedigree relationship to a target population selected by the selection criteria from multiple bi-parental families (DS2) for different subset sizes

Selection criteria	Subset size	Pop3	Pop5	Pop10	Pop19	Pop37	Pop41
CDmean	50	0.92	0.85	0.84	0.68	0.85	0.83
Avg_GRM		0.86	0.90	0.92	0.75	0.89	0.84
CDmean	100	0.88	0.72	0.88	0.67	0.83	0.73
Avg_GRM		0.89	0.85	0.87	0.70	0.87	0.78
CDmean	200	0.86	0.34	0.85	0.66	0.82	0.65
Avg_GRM		0.88	0.31	0.88	0.64	0.85	0.69
CDmean	300	0.83	0.23	0.80	0.65	0.55	0.52
Avg_GRM		0.89	0.24	0.88	0.61	0.64	0.62
CDmean	400	0.76	0.17	0.76	0.64	0.45	0.41
Avg_GRM		0.86	0.18	0.88	0.65	0.51	0.43
CDmean	500	0.68	0.12	0.71	0.62	0.38	0.26
Avg_GRM		0.74	0.14	0.79	0.64	0.43	0.30
CDmean	600	0.61	0.11	0.64	0.59	0.34	0.21
Avg_GRM		0.62	0.12	0.67	0.62	0.36	0.21
CDmean	700	0.53	0.10	0.57	0.57	0.29	0.15
Avg_GRM		0.53	0.10	0.57	0.57	0.31	0.16
CDmean	800	0.47	0.08	0.51	0.54	0.27	0.14
Avg_GRM		0.47	0.09	0.51	0.52	0.27	0.15
CDmean	900	0.43	0.08	0.46	0.49	0.24	0.14
Avg_GRM		0.43	0.09	0.46	0.48	0.24	0.14
CDmean	1000	0.39	0.07	0.42	0.46	0.22	0.14
Avg_GRM		0.39	0.07	0.42	0.46	0.22	0.14

**Fig. 4:**
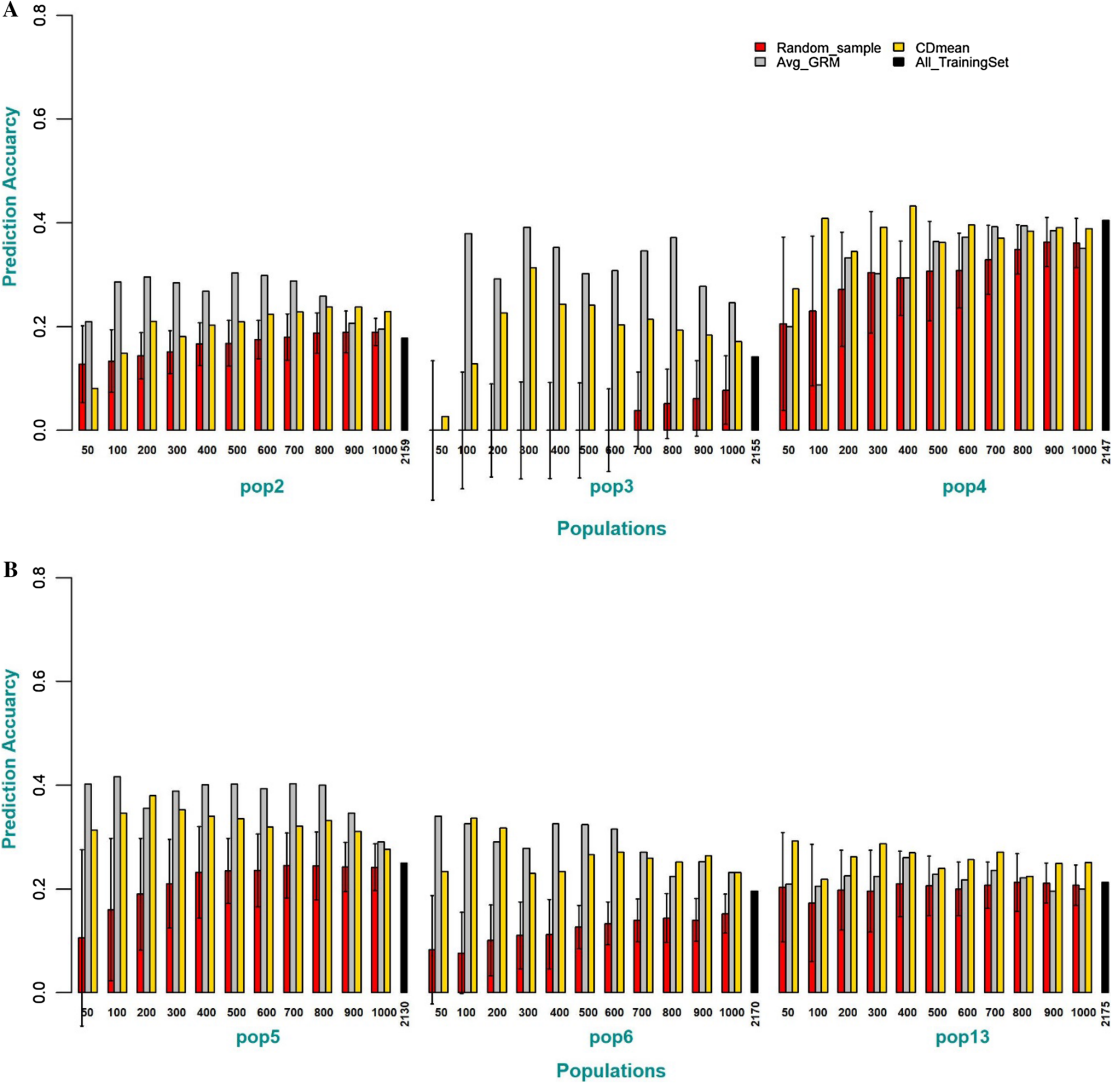
**a** Prediction accuracy of different training set sizes optimized using Avg_GRM and CDmean compared to random sampling of the training set or the combined DS1 and DS2 as a training set for populations 2, 3 and 4. For the random sampling (red color), the error bar represents the degree of variation in prediction accuracy of the 50 repetitions of random selection of individuals as the training set. **b** Prediction accuracy of different training set sizes optimized using Avg_GRM and CDmean compared to random sampling of the training set or the combined DS1 and DS2 as a training set for populations 5, 6, and 13. For the random sampling (red color), the error bar represents the degree of variation in prediction accuracy of the 50 repetitions of random selection of individuals as the training set

To further understand if increasing the size of the training set with individuals that have varying degrees of pedigree relationship was detrimental to prediction accuracy, the DS1 and DS2 dataset was combined to a single dataset. Since most populations in the DS1 dataset have half-sib pedigree relationship, each population was used in turn as BP. For all the subsets of the training set, the proportion of selected individuals that have a half-sib relationship with a target BP was greater than 60 percent for all the subset sizes and 75–90% for subsets of size 50 to 500 (Table 2). The prediction accuracy using the selection criteria was consistently higher than using all individuals as a training set or selection of the training set using random sampling. For instance, across all populations used as BP, a small optimized training set (100 individuals) formed using the selection criteria has higher prediction accuracy than using all individuals as the training population (2156, mean of TP across populations) (Fig. 4).

**Table 2:** The percentage of individuals with half-sib pedigree relationship to a target population selected by the selection criteria from combined DS1 and DS2 dataset for different subset sizes

Selection criteria	Subset size	Pop2	Pop3	Pop4	Pop5	Pop6	Pop13
CDmean	50	0.86	0.90	0.88	0.83	0.89	0.92
Avg_GRM		0.98	0.98	0.94	0.84	0.98	0.98
CDmean	100	0.88	0.88	0.86	0.79	0.86	0.85
Avg_GRM		0.96	0.92	0.88	0.83	0.95	0.85
CDmean	200	0.88	0.86	0.79	0.76	0.89	0.43
Avg_GRM		0.95	0.89	0.85	0.83	0.94	0.45
CDmean	300	0.88	0.86	0.76	0.74	0.87	0.30
Avg_GRM		0.95	0.87	0.78	0.83	0.94	0.30
CDmean	400	0.87	0.86	0.70	0.74	0.85	0.21
Avg_GRM		0.93	0.86	0.76	0.80	0.94	0.22
CDmean	500	0.842	0.84	0.70	0.68	0.84	0.18
Avg_GRM		0.93	0.86	0.76	0.79	0.95	0.18
CDmean	600	0.81	0.84	0.68	0.65	0.82	0.14
Avg_GRM		0.90	0.86	0.73	0.79	0.94	0.15
CDmean	700	0.79	0.82	0.68	0.62	0.76	0.13
Avg_GRM		0.87	0.82	0.72	0.75	0.92	0.13
CDmean	800	0.76	0.74	0.66	0.62	0.74	0.11
Avg_GRM		0.75	0.83	0.69	0.72	0.84	0.11
CDmean	900	0.69	0.71	0.66	0.61	0.71	0.10
Avg_GRM		0.71	0.75	0.66	0.69	0.76	0.10
CDmean	1000	0.65	0.67	0.63	0.60	0.67	0.09
Avg_GRM		0.65	0.68	0.64	0.65	0.69	0.09

### Comparison of predictive ability using CDmean and random sampling for training set design in sparse testing and test-half-predict-half genomic selection strategies

**Fig. 5:**
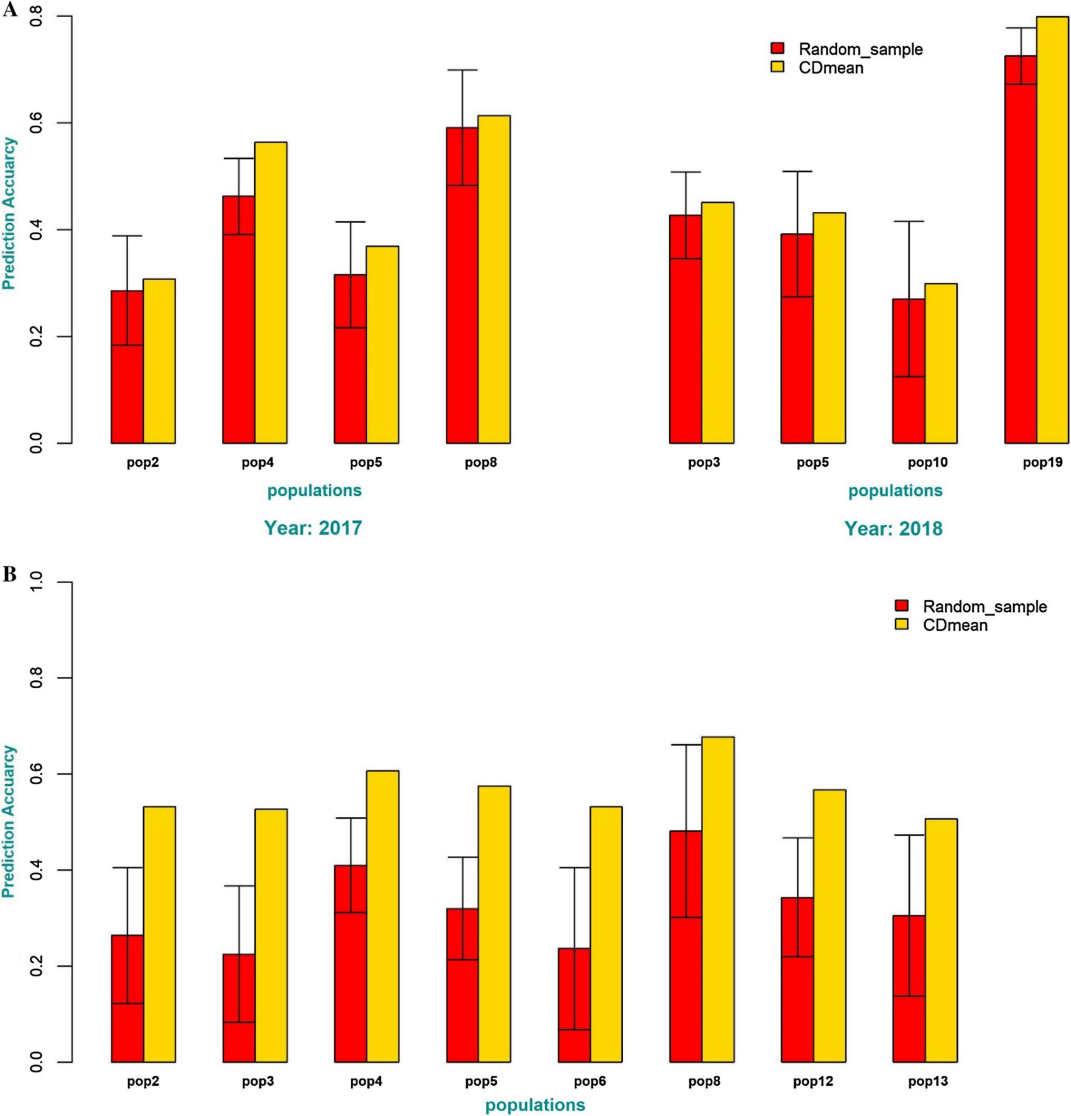
**a** The prediction accuracy of training set design by CDmean and mean of 50 repetitions of random sampling of training set in test-half-predict-half genomic selection strategy. The error bar is the variation in prediction accuracy of the 50 repetitions of random sampling of individuals in the training set. **b** The prediction accuracy of training set design by CDmean and mean of 50 repetitions of random sampling of training set in sparse testing genomic selection strategy. The error bar is the variation in prediction accuracy of the 50 repetitions of random sampling of individuals in the training set

The use of CDmean to identify individuals for field testing in a THPH scheme showed consistently higher prediction accuracy than the mean of the 50 repetitions random splits (50–50) in both GS strategies (Fig. 5). Figure 5a illustrates the consistency of improved prediction across populations in 2017 and 2018 using CDmean to choose the most representative individuals to be phenotyped. The prediction accuracy ranges from 0.31 to 0.61 using CDmean and 0.28 to 0.59 as mean of the 50 repetitions of random sample across the populations used in 2017, and 0.30 to 0.79 and 0.27 to 0.72 using CDmean and random sampling, respectively, for the populations considered in 2018. Further, in the sparse testing scheme, the prediction accuracy across all populations using CDmean ranges from 0.51 to 0.68, with the mean of the 50 repetitions of random sampling ranging from 0.23 to 0.48 (Fig. 5b).

### Accuracy when training set consists of full-sibs, half-sibs and half-sibs plus full-sibs

**Fig. 6:**
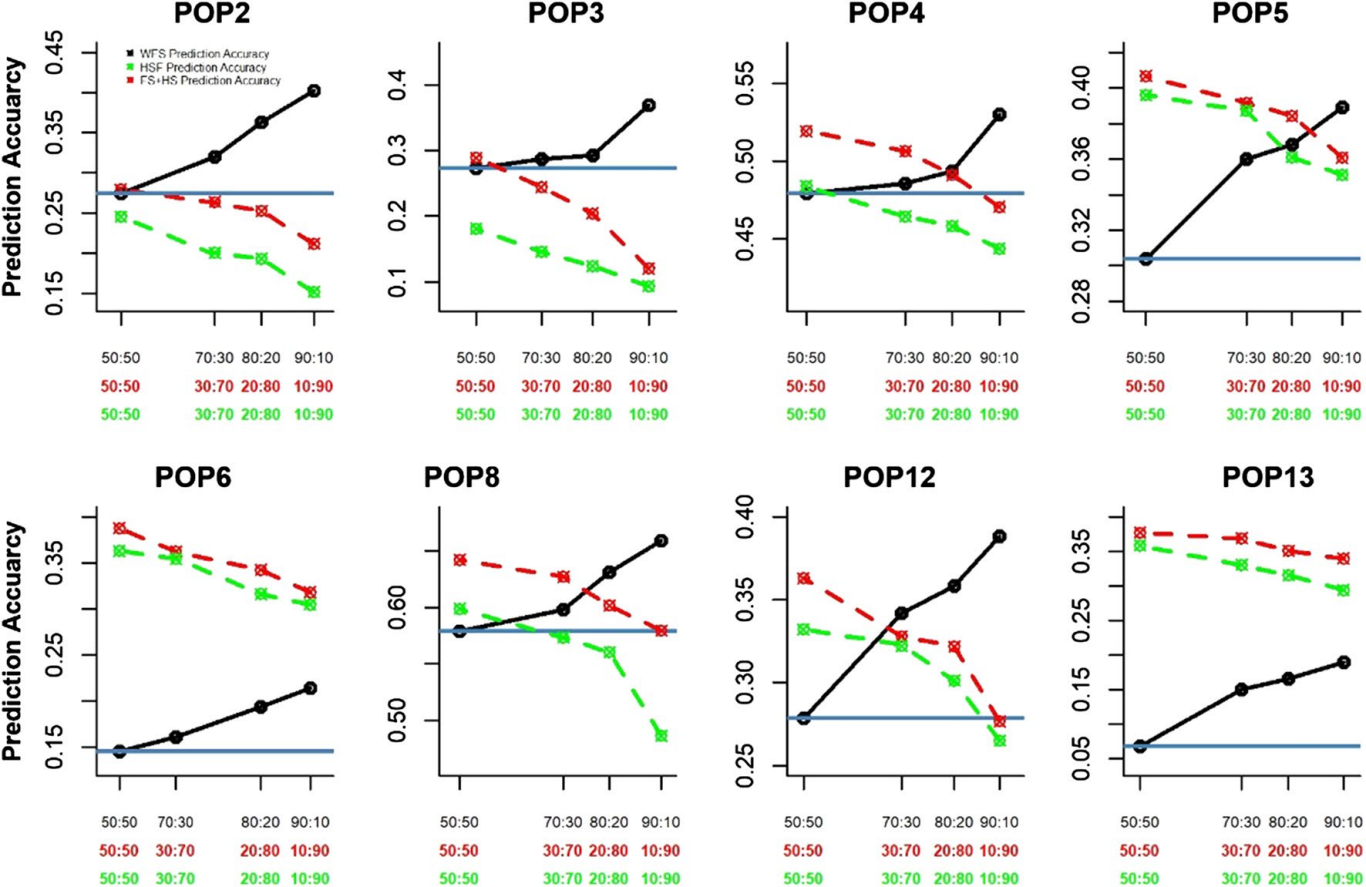
Prediction accuracy of eight full-sib populations with three different training sets: (i) full-sibs (black), (ii) half-sibs (green) and (iii) half-sibs plus full-sibs (red). Blue line is the prediction accuracy using 50 percent phenotyped and 50 percent predicted

THPH (50–50) using only FS data (Fig. 6 blue line) did not result in better prediction accuracy compared to using populations with half-sib pedigree relationship (HSF) (green lines) as training set except for population 2 and 3. Prediction accuracy using HSF plus full-sibs (FS) (red lines) as a training set was consistently high. Increasing the training set size for within FS (70:30, 80:20 and 90:10) (black lines) improves prediction accuracy and while decreasing the size of the HSF or HSF plus FS TPs (30:70, 20:80 and 10:90) lead to decline in the prediction accuracy. Overall, the use of all the HSF plus part of the FS as training results in higher prediction while use of only HSF is comparatively better than within FS prediction when the size of the family is small.

## Discussion

The main objective of this study was to develop and test prediction strategies for maximizing the advantages of GS in the CIMMYT global maize breeding program, particularly at the preliminary yield trial stage where it is expensive to phenotype large numbers of testcross hybrids in multiple locations. The focus was on identifying scalable methods that can be potentially deployed across diverse but genetically interlinked tropical maize breeding programs of CIMMYT which can help determine (1) if adequate prediction accuracy can be achieved for a given untested population using only information from historical data based on the representation of alleles in the historical data set; (2) how resources can be optimally assigned within the phenotyping network to improve prediction accuracy for any population where phenotyping some full sib progenies is needed; and (3) how to efficiently extract the most useful information from the historical data to maximize prediction accuracy across populations. The results of this study are relevant for the latter two of the above mentioned three objectives. We report the results of analyses of different methods to optimize prediction of previously untested bi-parental breeding populations with or without inclusion of FS data together with different strategies for designing TP using dynamic customized training sets.

For situations in which some or all FS progenies of a given bi-parental population must be phenotyped in a stage 1 trial (e.g., for populations with limited genetic connectivity to a robust historical data set), methods to improve either THPH or ST strategies were evaluated. Rincent et al. (2012) proposed optimal selection of lines to phenotype for model calibration to predict the GEBV of un-phenotyped lines using prediction error variance (PEVmean) and CDmean as optimization criteria. We demonstrate the utility of CDmean for improving prediction accuracy through optimal selection of which FS progenies to phenotype in a THPH scheme, and through optimal assignment of FS progenies to different environments in an ST scheme. The results indicate that, when implementing a THPH or ST approach, careful consideration should be given to which lines are phenotyped in which locations as it will impact the accuracy of predictions. Unlike traditional experimental designs, approaches that use genomic information need to balance the representation of parental chromosomal segments across environments. As these two schemes are not exclusive, optimized ST would be beneficial to the breeding program whether or not it is coupled with optimized THPH strategy, although this has not been assessed.

The incorporation of information from close relatives beyond the FS population should improve prediction accuracy since the degree of genomic relationship between individuals in BP and TP has been widely reported to be a major driver of genomic prediction accuracy (Habier et al. 2007; Clark et al. 2012; Taylor 2014; Lee et al. 2017). The utility of highly diverse historical data for genomic prediction is dependent on several factors including LD decay, marker density and genotype by environmental interactions (Goddard et al. 2011; Burgueño et al. 2012; Khansefid et al. 2014; Jarquín et al. 2014; Santantonio et al. 2020). In many situations, efficient extraction of the most informative individuals from existing genotypic and phenotypic data to calibrate models that predict the genetic merit of a target BP can be important for optimal use of historical breeding information in GS. This is demonstrated by the improved predictive ability of the CTS of size 200 to 600 selected by the optimization criteria when compared with random sampling or when using all available records as the TP, even though the dataset used in this study contained many highly related lines tested in similar environments. Further validation is required for historical data with genetically diverse lines tested across multiple environments. The selection criteria were efficient in accounting for relatedness, as they tended to select individuals that have half-sib pedigree relationship to the target BP present in the DS2 and in the combined data (DS1 and DS2) as part of the CTS.

Results from the combined analysis indicate that it is possible to achieve moderate prediction accuracy using data with varying degrees of genetic relationship and tested in different environments to train prediction models by using efficient optimization criteria to extract individuals from the combined dataset with the most predictive ability of the BP. When using markers sets of moderate density, as was the case in this study, the increased prediction accuracy in CTS is likely due to improved marker-QTL linkage phase between CTS and BP. Unfiltered historical data may have large independent segregating effective chromosome segments which contribute to lower prediction accuracy as was the case when using the combined datasets to predict populations in DS1. This suggests that unique QTL alleles and differences in marker-QTL linkage phase in unrelated populations could reduce the signal to noise ratio when predicting trait values for individuals from a given BP. Results of the current study corroborate earlier studies indicating that the genetic relationship between TP and BP strongly affects prediction accuracy (Andreescu et al. 2007; Crossa et al. 2010; Pszczola et al. 2012; Rincent et al. 2012; Taylor 2014; Hickey et al. 2014; Schopp et al. 2017). Validation of algorithms which can be used to routinely extract optimal CTS on a population by population basis is an important step toward developing a scalable pipeline to enable diverse, but interconnected breeding programs to efficiently use the most relevant available data from large shared data sets to increase prediction accuracy.

The relatively low prediction accuracy within each bi-parental population when using only FS progenies was likely the result of the small TP size on prediction accuracy. Multiple studies (Crossa et al. 2010; Rincent et al. 2012; de los Campos et al. 2013; Hickey et al. 2014; Schopp et al. 2017) indicate that given moderate marker density, prediction accuracy within a bi-parental population is driven by the size of the TP and the trait heritability. The small TP when using only FS progenies resulted in larger variation in prediction accuracy resulting from less consistent estimation of QTL effects, and this is reflected in the inflated standard error of the prediction accuracy. Hickey et al. (2014) and Schopp et al. (2017) also reported low prediction accuracy and high variation in prediction accuracy when they randomly sampled lines within bi-parental populations for cross-validation using TP size of less than 100.

While the number of FS progeny in the TP has a significant impact on prediction accuracy, the inclusion of HSF was always as good as or better than using FS progeny alone. The improved prediction accuracy obtained when HSF were included with FS progenies to develop training sets indicates that THPH approaches should incorporate full-sib information when possible, especially when the bi-parental populations are small. Inclusion of a small number of FS progenies from the target BP to complement the HSF in the TP results in higher prediction accuracy since extensive chromosome segments segregate within bi-parental population and the effects are better estimated. This indicates that phenotyping even a small number of full-sibs can be beneficial when historical data are sparse.

One of the critical factors in GS is the interpretation of prediction accuracy at every breeding stage, considering the distinctiveness or objective at each stage. For instance, traditionally at the preliminary/stage 1 yield trial, phenotypic selection (PS) is done using the estimated best linear unbiased predictor (BLUP) or Best Linear Unbiased Estimator (BLUE) to eliminate lines with poor genetic merit from advancing to multi-location yield trials. Several studies (Jacobson et al. 2014; Bassi et al. 2016; Bernal-Vasquez et al. 2017) point toward reduction in the selection cycle time as the primary advantage of GS compared with PS since PS accuracy is expected to be higher than GS accuracy. On the contrary, Beyene et al. (2019) reported similar selection accuracy for GS and PS when advancing lines from stage 1 to stage 2 yield trials. In early stage testing, plot-based heritability is typically low and calculated estimates of genetic merit are less accurate, because the performance of the lines are evaluated in few locations (Endelman et al. 2014; Cobb et al. 2019). Improvement of PS accuracy in stage 1 yield trials can be achieved by increasing the number of locations; however, this is constrained by limited resources. Additionally, the number of testing locations is not linearly related to heritability, and therefore genetic gain does not scale linearly with heritability (Endelman et al. 2014; Cobb et al. 2019).

As shown in Beyene et al. (2019), when the objective is to discard lines with poor genetic merit from advancing to resource demanding multi-location yield trials, moderate genomic prediction accuracy should suffice without losing selection accuracy. Prediction accuracy does not decline drastically when enough individuals close to the BP are in the TP as demonstrated in this study. This implies phenotyping at stage 1 yield trial can be eliminated using optimized TP without losing selection accuracy in advancing good performing lines to the resource demanding multi-location yield trials saving phenotyping costs and reducing selection cycle time, ultimately increasing genetic gain. The use of GS for stage 1 trial prediction coupled with ST could result in significant increases in breeding program efficiency without significant increasing breeding program costs, with savings from reduced replications of lines for phenotyping offsetting increases in genotyping cost.

Additionally, for breeding programs where past training information is not accessible, breeders may reduce the cost of phenotyping by evaluating connected bi-parental populations sharing one parent in common by phenotyping only a small number of lines from each population. This study suggests that prediction accuracy can be significantly improved compared to within-family prediction alone, especially when TP for within-population is small. Finally, it is shown that further improvement in prediction accuracy in either ST or THPH GS strategy can be achieved by optimizing the assignment of individuals planted in each environment and/or the individuals phenotyped and those that are evaluated based on genomic predictions.

## Conclusion

Routine implementation of GS in a plant breeding program must account for multiple factors including cost, logistics, accuracy and breeding schemes. In early stages of implementation, the lack of large historical training datasets presents additional challenges. When utilizing cost effective, low to mid-density genotyping platforms, leveraging family structure in breeding programs becomes increasingly important for achieving high accuracy. In such cases, the importance of training set design cannot be overemphasized. Here, we have demonstrated that the CIMMYT tropical maize breeding program can effectively utilize limited historical data when the TP is carefully selected to leverage population structure and family relationships. For populations with close relatives in historical datasets, phenotyping of lines at preliminary/stage 1 yield trials can be eliminated, using only GEBV to advance new lines into multi-tester multi-location yield trials. When working with populations having limited family relationships to historical data, approaches such as selective testing of subsets of the population in preliminary trials can be used to reduce the cost of early stage testing. As historical data are accumulated these two strategies will converge with the ultimate goal of eliminating all preliminary yield trials, reducing the time required to develop new varieties and reducing generation intervals by enabling earlier recycling of new inbred lines. Given it is unlikely to have a single large TP with high predictive ability across BP, algorithms such as CDmean or Avg_GRM will be required to select BP specific CTS from historical datasets. For populations that require targeted phenotyping of FS, algorithms such as CDmean should be employed to select individuals for phenotyping, and phenotypic records from closely related populations can be leveraged to significantly improve prediction accuracy. Though our emphasis is on CIMMYT tropical maize breeding program, the conclusions drawn from our study should apply to other public maize breeding programs implementing GS.

## Electronic supplementary material

Below is the link to the electronic supplementary material.

Supplementary material 1 (PDF 443 kb)
