# Supplementary material for: Maximizing efficiency of genomic selection in CIMMYT’s tropical maize breeding program
Source: Theor Appl Genet. 2020 Oct 10;134(1):279–94. doi: 10.1007/s00122-020-03696-9 (PMC7813723; doi:10.1007/s00122-020-03696-9)
Supplement: Supplementary file 1 — Supplementary material 1 (PDF 443 kb) [file 122_2020_3696_MOESM1_ESM.pdf]

Online Resources 1: Pedigree and size of 13 biparental doubled-haploid families in 2017

Population	Size	Parent1	Parent2
pop1	30	CML440	La Posta Seq C7-F64-2-6-2-2-B-B
pop2	87	CML445	La Posta Seq C7-F64-2-6-2-2-B-B
pop3	83	CML444	La Posta Seq C7-F64-2-6-2-2-B-B
pop4	91	CML312	La Posta Seq C7-F64-2-6-2-2-B-B
pop5	108	CML442	La Posta Seq C7-F64-2-6-2-2-B-B
pop6	68	CML505	La Posta Seq C7-F64-2-6-2-2-B-B
pop7	44	CZL04003	La Posta Seq C7-F64-2-6-2-2-B-B
pop8	88	CML536	La Posta Seq C7-F64-2-6-2-2-B-B
pop9	53	CML537	La Posta Seq C7-F64-2-6-2-2-B-B
pop10	37	CML538	La Posta Seq C7-F64-2-6-2-2-B-B
pop11	31	INTA-F2-192-2-1-1-1-B*7-3-B-B	La Posta Seq C7-F64-2-6-2-2-B-B
pop12	66	ZEWAc1F2-134-4-1-B-1-B*4-1-2-B-B	La Posta Seq C7-F64-2-6-2-2-B-B
pop13	63	CML312	INTA-F2-192-2-1-1-1-B*7-2-B-10-B-B-B

Online Resources 2: Pedigree and size of 45 biparental doubled-haploid families in 2018

Population	Size	Parent1	Parent2
pop1	24	CKDHL0159	CML202
pop2	39	CKDHL0221	CKDHL120312
pop3	84	CKDHL0228	CKDHL120312
pop4	47	CKDHL120143	CKDHL120312
pop5	60	CKDHL120143	CKDHL121167
pop6	45	CKDHL120184	CKDHL120312
pop7	43	CKDHL120312	CKDHL0214
pop8	39	CKDHL120312	CKDHL121167
pop9	20	CKDHL120312	CML312
pop10	53	CKDHL120312	CML464
pop11	16	CML144	CML202
pop12	27	CML202	CKDHL0089
pop13	27	CML202	CKDHL121167
pop14	31	CML202	CKL05003
pop15	39	CML206	CML464
pop16	27	CML322	CML202
pop17	46	CML463	CKDHL120312
pop18	44	CML463	CML536
pop19	91	CML536	CKDHL120312
pop20	9	CML540	ZEWAc2F2-183-2-BBB-B-B-B-B
pop21	10	CML545	CML540
pop22	14	ZEWAc1F2-134-4-1-B*	PHG29

pop23	19	ZEWAc1F2-300-2-2-B*	LH119
pop24	9	ZEWAc1F2-300-2-2-B*	LH51
pop25	10	ZEWAc1F2-300-2-2-B*	PHG29
pop26	3	[(CML395/CML444)-B-4-1-3-1-B/CML395//DTPWC8F31-1-1-2-2]-5-1-2-2-BB-B-B-B	CML507
pop27	40	2369/CML536	CML539
pop28	36	CML509	CML312
pop29	27	LH123HT/CML539	CML539
pop30	45	PB80/CML536	CML539
pop31	48	(PHW52/[MSRXG9]C1F2-205-1(OSU23i)-5-3-X-X-1-BBB-1-B*7	CML539
pop32	31	RS_CKSBL10004	CML442
pop33	6	RS_CKSBL10020	CML444
pop34	6	RS_CML395	CZL00003
pop35	11	RS_CML444	CKSPL10073
pop36	10	RS_CML444	CZL03014+
pop37	49	TZMI711	CML539
pop38	44	ZEWAc2F2-152-1-BBB-B-B-B-B	CML540
pop39	46	ZEWAc2F2-183-2-BBB-B-B	00SADVEA-#-28-1-2-1-1-1-2-3-B
pop40	60	PHG39/CML539	CML539
pop41	1	CKDHL121167	CML464
pop42	1	CKSBL10004	NA
pop43	1	CML312	NA
pop44	1	CML395	NA
pop45	1	CML442	NA

+ the same as CML539

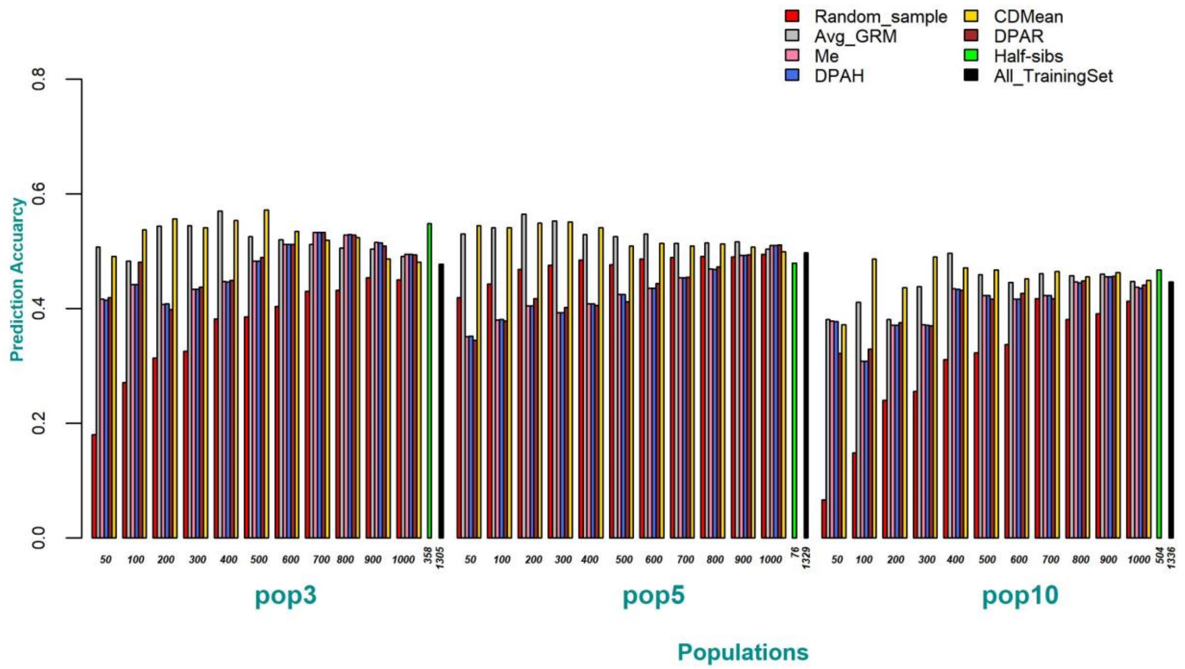

Online Resources 3A: Prediction accuracy of several training set size optimized using different optimization criteria compared to random sampling of the training set and use of all the populations (MBP) as training set

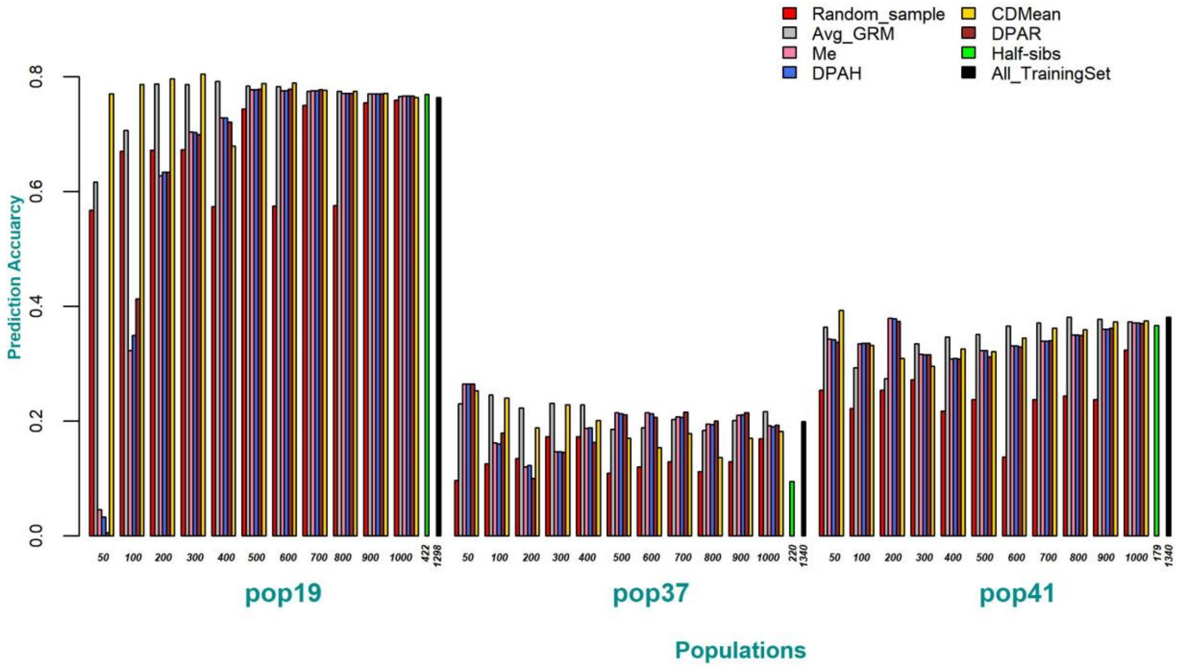

Online Resources 3B: Prediction accuracy of several training set size optimized using different optimization criteria compared to random sampling of the training set and use of all the populations (MBP) as training set
